# In silico functional and structural characterization of hepatitis B virus PreS/S-gene in Iranian patients infected with chronic hepatitis B virus genotype D

**DOI:** 10.1016/j.heliyon.2020.e04332

**Published:** 2020-07-15

**Authors:** Nastaran Khodadad, Seyed Saeed Seyedian, Afagh Moattari, Somayeh Biparva Haghighi, Roya Pirmoradi, Samaneh Abbasi, Manoochehr Makvandi

**Affiliations:** a Infectious and Tropical Disease Research Center, Health Research Institute, and Department of Virology, Ahvaz Jundishapur University of Medical Sciences, Ahvaz, Iran; b Alimentary Tract Research Center, Ahvaz Jundishapur University of Medical Sciences, Ahvaz, Iran; c Department of Bacteriology and Virology, Shiraz University of Medical Sciences, Shiraz, Iran; d Department of General Courses, School of Medicine, Ahvaz Jundishapur University of Medical Sciences, Ahvaz, Iran; e Abadan Faculty of Medical Sciences, Abadan, Iran

**Keywords:** Bioinformatics, Immunology, Microbiology, Genetics, Molecular biology, In silico, Chronic hepatitis B, Mutations, Iran

## Abstract

**Objective:**

Chronic hepatitis B (CHB) virus infection is the most prevalent chronic liver disease and has become a serious threat to human health. In this study, we attempted to specify and predict several properties including physicochemical, mutation sites, B-cell epitopes, phosphorylation sites, N-link, O-link glycosylation sites, and protein structures of S protein isolated from Ahvaz.

**Materials and methods:**

Initially, hepatitis B virus DNA (*HBV* DNA) was extracted from five sera samples of untreated chronic hepatitis B patients. The full-length *HBV* genomes were amplified and then cloned in pTZ57 R/T vector. The full sequences of *HBV* were registered in the GenBank with accessions numbers (MK355500), (MK355501) and (MK693107-9). PROTSCALE, Expasy's ProtParam, immuneepitope, ABCpred, BcePred, Bepipred, Algpred, VaxiJen, SCRATCH, DiANNA, plus a number of online analytical processing tools were used to analyse and predict the *preS/S* gene of genotype *D* sequences. The present study is the first analytical research on samples obtained from Ahvaz.

**Results:**

We found major hydrophilic region (MHR) mutations at “a” determining region that included *K122R, N131T, F134Y, P142L,* and *T126N* mutations. Moreover, Ahvaz sequences revealed four sites (4, 112, 166, and 309) in the *preS/S* gene for N-glycosylation that could possibly be a potential target for anti-*HBV* therapy.

**Conclusion:**

In the present study, mutations were identified at positions T113S and N131T within the MHR region of S protein; these mutations can potentially decrease the effect of hepatitis B vaccination in vaccine recipients.

## 1 Introduction

Hepatitis B virus (HBV) infection remains as one of the major problems of health worldwide, with at least 257 million individuals who are chronically infected [1]. At present, the seventh leading cause of global death is viral hepatitis, with a 63% mortality rate which increased to 1,450,000 from 1990 to 2013 [2,3]. Annually more than 680 thousand people die due to cirrhosis and hepatocellular carcinoma (HCC) infected with HBV [1,2]. HBV is a small DNA virus belonging to the genus of *Orthohepadnavirus* and the family of *Hepadnaviruses* whose genome is relaxed, circular, and partially double-stranded, and about 3200 base pairs (bp) in length [4]. HBV has four partially overlapping open reading frames (ORF) that encode the core (preC/C), envelope (preS/S), polymerase (Pol) and X proteins. The envelope protein of HBV contains small, middle and large hepatitis B surface antigen (HBsAg) proteins, which are encoded by S (preS1/preS2/S) ORF [2]. These proteins are employed for attaching to hepatic cells (S), contain several epitopes of B and T cell (M), and are used for diagnosing HBV infections in individuals (L) [2,5,6]. Based on the presence of amino acid at positions 122 and 160, four serotypes (adw, ayw, adr, and ayr) are accessible. Also, based on the existence of amino acid at positions 127, 134 and 159, HBV create nine subtypes (adw2, adw4, adrq+, adrq−, ayw1, ayw2, ayw3, ayw4, and ayr) which are useful as epidemiologic markers [5,7]. To date, concerning the nucleotide divergence >7.5% of the viral genome, HBV has been classified into 10 genotypes (A-J). Subgenotypes have been identified by the intergroup nucleotide divergence of 4–8 % among genotypes [2,5].

The major antigenic domains of HBV is located in the envelope proteins. In particular, the amino acids 99–170 of the S protein is termed major hydrophilic region (MHR) which contains a critical epitope exposed on the surface antigen of the virus [8]. The occurrence of mutations in MHR N-glycosylation may influence viral characteristics. The ‘‘a’’ determinant is an approximately conserved region (124–147 a.a) within the MHR that is the main key of humoral responses [9,10]. The HBsAb may be produced if the HBsAg clearance in a patient with acute infection occurs [11]. Protective antibodies (anti-HBs) create a complex with free viral particles and result in removing viruses from blood circulation and preventing the attachment of HBV to hepatocytes. The majority of HBsAg tests use antibodies that target residues located between amino acids 100–160 to detect the existence of the HBsAg. Consequently, mutations occurred in the ‘a’ determinant domain that localize to this target section can result in the false-negative test outcomes [8]. G145R is a common immune escape mutation that is induced by the vaccine and can lead to HBsAg detection failure by some tests. Also, mutations may occur in association with either vaccine-induced immune-escape (P120T, K122R, T126S, Q129H, G130N, M133L, and M133T) or in relation to the patients with occult HBV infection (Y100C, C124R, C124Y, K141E, and D144A) [8,10].

The nucleotide substitutions in the pre-S1 promoter region, particularly the G2765A mutation, reduce the L protein expression and regulate the biological situation of HBV and result in a low viral load and then less serious disease in chronic HBV infection [12]. The mutations in pre-S2 may affect the signalling of the HBV-infected cells and increase tumorigenesis [13]. Mutations in pre-S regions have caused a decrease of binding affinity of monoclonal antibodies facing HBsAg and ended in the low expression of HBsAg. These data not only indicate the importance of pre-S regions which can induce neutralizing antibodies but also approve that alterations may be associated with the development of liver diseases [14]. World Health Organization (WHO) has planned to eradicate HBV and HCV infections worldwide by 2030. For this purpose, the sequence analysis of HBV DNA isolated from patients with chronic hepatitis (CHB) and occult hepatitis (OBI) should be determined in different regions of the world [2]. Our study attempted to determine the major variations in the preS/S gene isolated from patients with chronic hepatitis B virus infection, genotype D in Ahvaz, Iran. Based on the bioinformatics data, the characterization of preS1, preS2 and S proteins of the isolates belonging to HBV genotype D1 was required for the evaluation completed in the present research.

This study attempted to identify mutations (*K122R, N131T, F134Y, P142L,* and *T126N*) in S gene, N-glycosylation sites (4, 112, 166, and 309), compare Ahvaz samples with isolates from other regions of the world using the phylogenetic tree, and investigate the tertiary structures of isolates. Ramachandran plot showed that most residues were placed in the favoured regions rather than allowed regions. All isolates of Ahvaz had genotype D and sub-genotype D1. Mutations in the MHR region can reduce the effect of vaccination on vaccine recipients. In the present study, no G145R mutation was detected in the S protein of both HBV isolates from Ahvaz.

## 2 Material and methods

### 2.1 Sampling

Sera samples were collected from five untreated patients (48.6% from females and 51.4% from males) with chronic hepatitis B virus infection (serum HBsAg positive for at least six months) who referred to Ahvaz hospitals during 2018. The patients ranged from 33 to 70 years of age, with the mean age of 47.2 years; all patients were married. All samples were tested for HBsAg, HBeAg, HBcAb, and HBeAb using ELISA kits (DiaPro, Italy) according to the manufacturers’ protocols.

### 2.2 Ethical statement

This project was approved with ethical code IR.AJUMS.REC.1397.326 and registration number OG-9723 by the ethics committee of Ahvaz Jundishapur University of Medical Sciences. The ethic consent was obtained from all individuals participated in this survey. The study was carried out in accordance with the Declaration of Helsinki.

### 2.3 DNA extraction & full-length amplification

To analyse the preS/S gene of HBV in this study, viral DNA was extracted by High Pure Viral DNA Extraction Kit (Roche, Germany) from serum according to the manufacturer's instructions. Full-length HBV genomes were amplified with a pair of primers (full HBV) previously described by Günther et al. [15]. The amplification conditions consisted of an initial denaturation step of 2 min 30 s at 95 °C, followed by 35 cycles of 1 min at 94 °C, 1 min 30 s at 50 °C, and 3 min 15 s at 72 °C and final extension of 10 min at 72 °C. The PCR reaction was carried out by using a 2X master mix PCR which included Taq polymerase (amplicon, Denmark) and added 3ʹ A overhangs in the end of HBV DNA sequences used in TA cloning. PCR products were analyzed by electrophoresis on 1% agarose gels, stained with Safe-Red and observed under UV light.

### 2.4 TA cloning and sequencing of full-length HBV genomes

PCR products of 3.2 kb were extracted from the catted agarose gels using GF-1 PCR clean-up kit (Vivantis, Malaysia). Extracted DNAs were cloned into the pTZ57 R/T vector (Thermo Fisher, USA) and then transformed into competent cells containing *Escherichia coli* (*E.coli*) strain DH5α using by Calcium Chloride method [16]. The transformed cells were incubated at 37 °C for 18 h and then clones per sample were confirmed by colony PCR with M13 specific primers. The cloned full-length *HBV* was sequenced by Microsynth (Balgach, Switzerland) with BigDye Terminator chemistry (v3.1), using four primers shown in (Supplementary material, TableI).

### 2.5 Analysis of HBV sequences

In this study, the obtained five sequences of full-length HBV were deposited in GenBank with accession numbers MK355500, MK355501 and MK693107-9. Concerning the high similarity of these sequences, we selected two sequences and performed the bioinformatics analysis on them. To evaluate the characterization of the detected preS/S gene, bioinformatics analysis was carried out. In addition to the two sequences, we selected 31 other sequences from GenBank and accession number NC003977.2 from USA were used as the reference sequence (RefSeq) (Supplementary material, TableII).

### 2.6 Amino acid changes

Amino acid mutations in the S gene of HBV may affect the immunogenicity and antigenicity of HBsAg, which result in immune escape and failure of viral diagnostic procedures [17]. For the analysis of mutations in all sequences of the preS/S gene, translation and editing were performed by BioEdit [18] and SnapGene (GSL Biotech LLC at http://www.snapegene.com) software [19]. The codons of ORF (S, preS1, and preS2) were analysed in sequences of patients with chronic HBV infection. The positions of codon were numbered from the beginning of each ORF. The CLC sequence viewer6 software and online tool Geno2pheno accessible at https://hbv.geno2pheno.org/ were used to respectively identify amino acid mutations and immune escape mutations [20].

### 2.7 Phylogenetic tree

Phylogenetic tree explains the relationship between genes, proteins, species or other taxa. It can also indicate recombination and evolutionary history of related organisms. In this research, the proteins alignment of entire sequences of preS/S gene was produced using ClastalW2 program [21] in the MEGA6 software [22]. The phylogenetic tree was constructed by a maximum-likelihood method and Tamura-Nei model for the preS/S gene (1000 replicates). Evolutionary distances were calculated based on Gamma distributed method with Discrete Gamma Categories 5.

### 2.8 Analysis of primary sequences

The online ExPASy ProtParam tool [23] available at http://expasy.org/tools/protparam.html was employed for the analysis of entire preS/S gene including calculating molecular weight, theoretical isoelectric point (PI), extinction coefficient, aliphatic index, instability index, grand average of hydropathy (GRAVY) and the total number of positive and negative residues. The Aliphatic index always correlates with amino acids which possess aliphatic side chains (A, V, I and L) and defines thermostability of proteins. This index is related to the content of the globular protein of the preS/S gene which influence unfolding and folding processes. Polarity, hydrophobicity, refractivity, bulkiness, recognition factors, percent accessible residues, percent buried residues, average flexibility, average area buried, transmembrane tendency, the number of codons and amino acids, and relative mutability were evaluated by the online ProtScale tool accessible at http://us.expasy.org/tools/protscale.html [24,25].

### 2.9 Analysis of immuno-informatics

B cell epitopes prediction of preS/S gene was determined by using online tool at www.immuneepitope.org (http://tools.immuneepitope.org/tools/bcell/iedb_input) [26]. This server uses the following methods: Pellequer and Westhof method [27] used for predicting Turns, Parker method [28] for hydrophilicity evaluation, Emini method [29] for surface accessibility and Karplus and Schulz method [30] for predicting the flexibility. Bepipred software at http://www.cbs.dtu.dk/services/BepiPred/ was also employed to predict Linear B cell epitopes [31]. To detect the polarity-based B cell epitopes, BcePred software at http://www.imtech.res.in/raghava/bcepred was run on the S gene sequences [32]. Moreover, B cell epitopes were predicted by using ABCpred server at http://www.imtech.res.in/raghava/abcpred/ [33]. VaxiJen server (available at http://www.ddg-pharmfac.net/vaxijen/VaxiJen/VaxiJen.html) was also used to estimate the probability of antigenicity. Default threshold in this software was determined 0.4 [34]. Finally, AlgPred software was operated to predict IgE epitopes at http://www.imtech.res.in/raghava/algpred/submission.html [35].

### 2.10 Functional characterization

DiANNA server available at http://clavius.bc.edu/∼clotelab/DiANNA/ [36], and SCRATCH at http://scratch.proteomics.ics.uci.edu/ [37] were used to predict disulfide bonds positions. Phosphorylation sites of serine, tyrosine and threonine in eukaryotic proteins were predicted by using online tools such as DISPHOS at http://www.dabi.temple.edu/disphos/pred.html [38,39] and NetPhos at http://www.cbs.dtu.dk/services/NetPhos/ [40]. GlycoEP at http://www.imtech.res.in/raghava/glycoep/submit.html [41] and NetNGlyc at http://www.cbs.dtu.dk/services/NetNGlyc/ [42] were used to predict N-glycosylation sites.

### 2.11 Secondary structure prediction

Secondary structure of overall sequences were predicted by using SOPMA software at http://npsa-prabi.ibcp.fr/cgi-bin/npsa_automat.pl?page=npsa_sopma.html [43,44]. GAMMAPred software at http://www.imtech.res.in [45] was used to confirm the results.

### 2.12 Tertiary structure prediction and validation

The overall 3D sequences of S gene were created in Phyre2server at http://www.sbg.bio.ic.ac.uk/phyre2/html [46], I-TASSER at http://zhanglab.ccmb.med.umich.edu/I-TASSER [47] and (PS)_2_–V2 Server at http://ps2v2.life.nctu.edu.tw [48]. The quality and stereochemistry of the models were evaluated via the online tool Qmean [49] available at http://swissmodel.expasy.org/qmean/cgi/index.cgi. Rampage (http://mordred.bioc.cam.ac.uk/rapper/rampage.php) [50] was also used to draw the Ramachandran plots.

## 3 Results

### 3.1 Phylogenetic analysis

**Figure 1:**
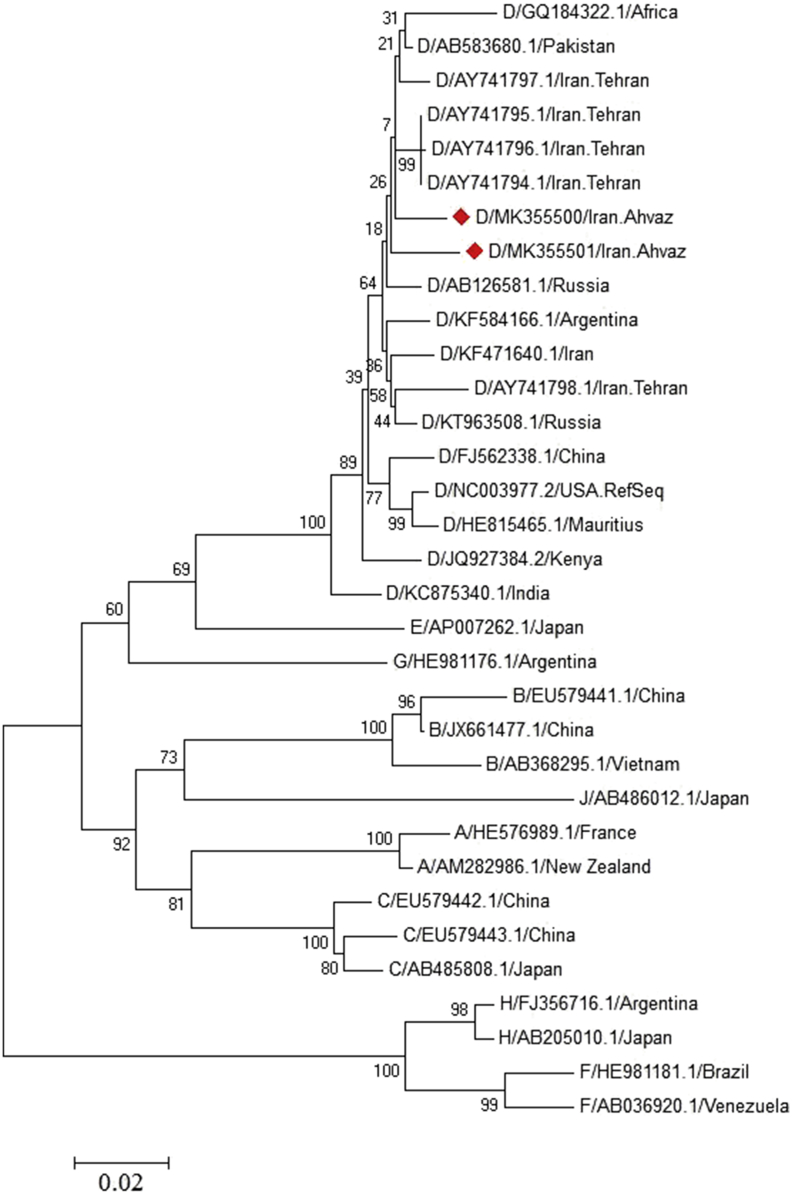
The phylogenetic tree was constructed using maximum likelihood method and Tamura-Nei model. The preS/S gene among HBV isolates with accession number (MK355500, MK355501) are retrieved from Ahvaz (represent with red diamond) in cluster with other HBV genotype D isolates from different regions of the world. The Bootstrap value was 1000 replicates. The scale bar indicates 2% nucleotide sequence divergence.

The sequences of S gene including 1170 base pairs and 389 amino acids were analysed by SnapGene software. To build the phylogenetic tree for the 33 HBV isolates of this study, maximum likelihood was operated (see Figure 1). The obtained results showed that the isolates from Ahvaz (MK355500, MK355501) were very closely related with the sequences of D1 genotype of other isolates from different regions of the world.

### 3.2 Amino acid changes

**Figure 2:**
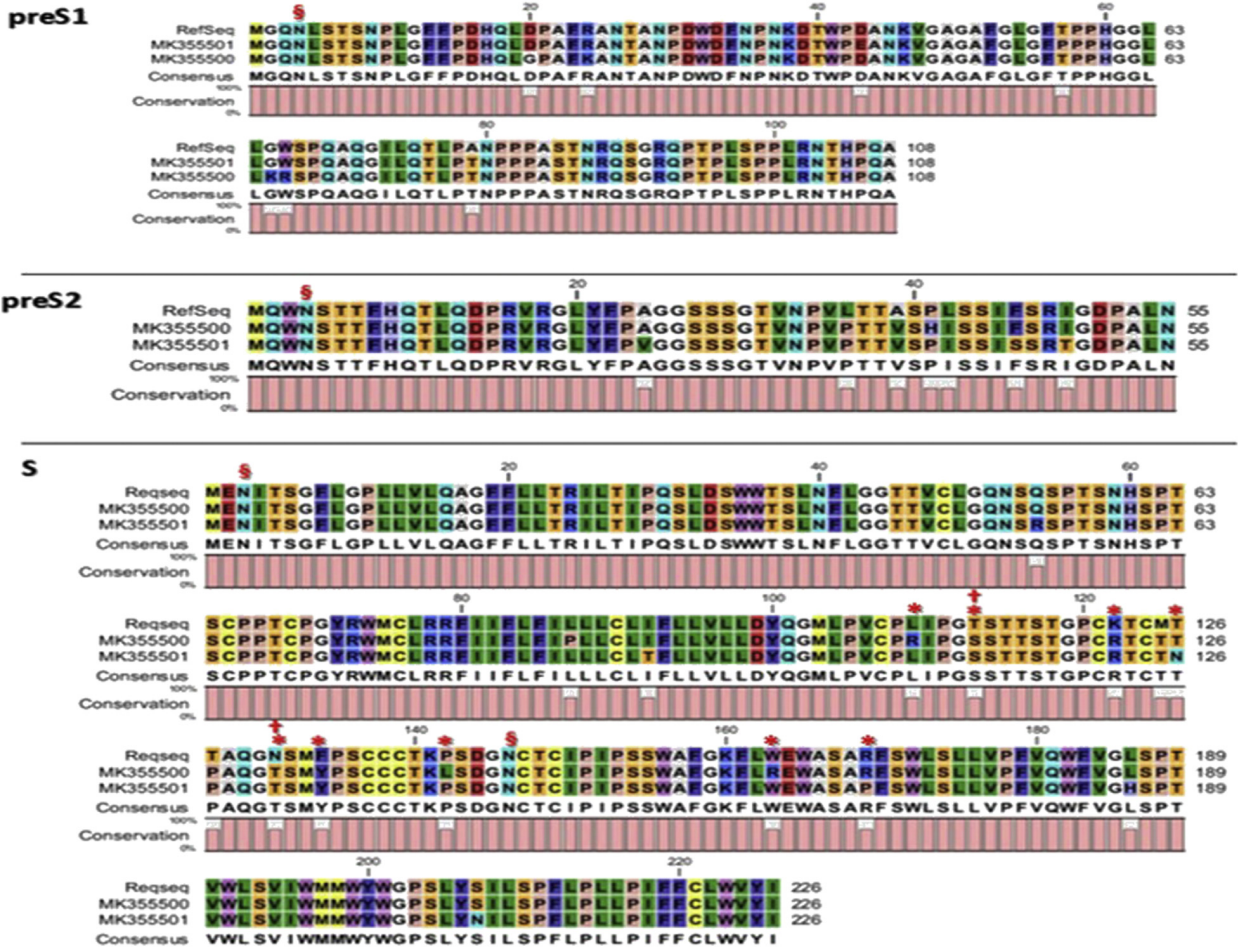
Amino acid changes in preS1/preS2/S regions of Ahvaz isolates and RefSeq using by CLC Sequence software. §: N-glycosylation site; ∗: Occurred mutations at MHR region; and †: Immune escape mutations.

The altered conformation of the protein occurred by the mutation on the S gene in various ways. Comparison analysis for Ahvaz sequences and RefSeq showed multiple amino acid substitutions on preS/S gene (Figure 2, and supplementary material, TableIII). The results of our amino acid sequences of the preS/S gene compared to consensus genes showed several mutations at position L109R, T113S, K122R, T126N, N131T, F134Y, P142L, W163R and R169P of the major hydrophilic region (MHR).

### 3.3 Physicochemical properties

Protparam results are summarized in Table 1. Theoretical pI for two sequences of Ahvaz was 8.40–8.98 which determined that the S protein can be a basic (or alkaline) protein. The grand average of hydropathicity was almost similar in the Ahvaz isolates. Further, Protscale outcomes revealed similarity for Ahvaz sequences. SignalP and Signal-BLAST did not find any cleavage site for S protein in the Ahvaz isolates but predisi predicted a signal peptide in both sequences of Ahvaz (64aa and 62aa).

**Table 1:** Physicochemical properties of Ahvaz sequences predicted by ProtParam tool.

Properties	pI	Half-life in *E.coli*	Instability index	Class	Aliphatic index	GRAVY
RefSeq	8.40	10h	54.98	Unstable	82.24	0.146
MK355500	8.98	10h	54.81	Unstable	80.46	0.081
MK355501	8.40	10h	58.83	Unstable	78.95	0.065

### 3.4 Analysis of B cell epitopes

For the aim of this study, B-cell epitopes were predicted according to the Immune Epitope Database (IEDB). The obtained results are presented in Table 2. Accordingly, patterns of similarity emerged among Ahvaz sequence isolates and RefSeq concerning beta-turns, antigenicity, and flexibility properties. But these sequences exhibited different positions regarding hydrophilicity, and surface accessibility properties. Results of ABCpred, Bepipred, BcePred, AlgPred and VaxiJen are summarised in Table 3. ABCpred identified a start position (374) for 16 meric B-cell epitopes in both of Ahvaz isolates and RefSeq. Bepipred analysis for Linear B cell epitopes demonstrated conserved regions in two isolates of Ahvaz and RefSeq. BcePred identified a critical position for B-cell epitopes in the two selected sequences of this study in Ahvaz and RefSeq. Two major positions (60–66 and 321–332) were also detected only in MK355500 protein sequence. The analysis of AlgPred prediction showed that our sequences were non-allergen proteins. Based on the prediction by VaxiJen, all the proteins sequences were antigenic.

**Table 2:** Prediction of B cell epitopes performed by five methods of the Immune Epitope Database and Analysis Resource.

	Hydrophobicity prediction	Flexibility prediction	Accessibility prediction	Beta-Turn prediction	Antigenicity prediction
**RefSeq**	303–309 (TKPSDGN)	133–139 (GGSSSGT)	21–45 (PAFRANTANPDWD FNPNKDTWPDAN)	133–139 (GGSSSGT)	241–262 (RRFIIFLFILLLCLIFLLVLLD)
**MK355500**	133–139 (GGSSSGT)	133–139 (GGSSSGT)	23–45 (FKANTANPDWD FNPNKDTWPDAN)	133–139 (GGSSSGT)	241–262 (RRFIIFLFIPLLCLIFLLVLLD)
**MK355501**	303–309 (TKPSDGN)	133–139 (GGSSSGT)	21–45 (PAFRANTANPDWD FNPNKDTWPEAN)	133–139 (GGSSSGT)	241–262 (RRFIIFLFILLLCLTFLLVLLD)

The results showed the selected epitopes at positions 21–45, 23–45, 133–139, 241–262, 303–309 with high scores among the other epitopes for B cell prediction.

**Table 3:** Results of Bcepred, Bepipred, Algpred, ABCpred, and VaxiJen for Ahvaz sequences.

	Bcepred	Bepipred	ABCpred	VaxiJen	Algpred
RefSeq	236–242	4–166, 197–227, 271–297, 304–310	374	0.5333 Antigen	NON ALLERGEN
MK355500	60–66, 236–242, 321–332	4–166, 197–228, 266–297, 303–308	374	0.5066 Antigen	NON ALLERGEN
MK355501	236–242	4–166, 197–227, 272–297, 304–309	374	0.5383 Antigen	NON ALLERGEN

Based on the default threshold in Algpred, the scale below -0.4 indicates non-allergen protein so both isolates (MK355500, MK355501) from Ahvaz strain and RefSeq showed non-allergen proteins.

### 3.5 Functional analysis

Using DiANNA server, seven disulfide bonds were detected in all sequences but the result of SCRATCH were detected in six positions for disulfide bonds which were dissimilar in our selected sequences (Table 4). Based on the results obtained from DISPHOS, no phosphorylation site was detected in Ahvaz sequences and RefSeq but NetPhos detected several phosphorylation sites. Based on the prediction by NetNGlyc, 4 N-linked glycosylation sites (4, 112, 166 and 309) were found in our sequences. Also, 5 N-linked glycosylation sites (4, 112, 166, 222 and 309) were predicted by GlycoEP. To predict O-linked glycosylation sites, GlycoEP server was employed and many sites were found (see Table 5).

**Table 4:** Prediction of disulfide bond positions for Ahvaz sequences using SCRATCH and DiANNA servers.

Sequences	SCRATCH	DiANNA
RefSeq	211–232, 228–239, 284–301, 287–302, 300–310, 312–384	211–239, 228–232, 253–300, 270–384, 284–287, 301–310, 302–312
MK355500	211–232, 228–239, 284–301, 287–302, 300–310, 312–384	211–284, 228–384, 232–287, 239–270, 253–300, 301–310, 302–312
MK355501	228–239, 270–287, 284–301, 232–253, 300–310, 312–384	211–239, 228–284, 232–287, 253–300, 270–384, 301–310, 302–312

**Table 5:** Results of N-link and O-link glycosylation sites prediction using NetNGlyc and GlycoEP for our selected sequences.

	NetNGlyc	GlycoEP N-link	GlycoEP O-link
**RefSeq**	4, 112, 166, 309	4, 112, 166, 222, 309	6, 7, 76, 85, 86, 90, 95, 104, 135, 137, 139, 145, 146, 148, 190, 200, 209, 216, 220, 221, 224, 226, 227, 231, 276–281, 286, 289, 290, 294, 295, 303, 311, 352
**MK355500**	4, 112, 166, 309	4, 112 166, 222 309	6, 7, 27, 76, 79, 85, 86, 90, 95, 104, 135, 137, 139, 145, 146, 148, 190, 200, 209, 216, 220, 221, 224, 226, 227, 231, 276–281, 286, 288, 289, 294, 295, 303, 311, 352
**MK355501**	4, 112, 166, 309	4, 112 166, 222 309	6, 7, 76, 79, 85, 86, 90, 95, 104, 135, 137, 139, 145, 146, 148, 151, 152, 157, 190, 200, 209, 216, 220, 221, 224, 226, 227, 231, 276–281, 286, 288, 294, 295, 303, 311, 352

The high score of N-glycosylation at positions 309 and 4 were computed by NetNGlyc and GlycoEP N-link, respectively. Besides, GlycoEP O-link software determined the high score of O-glycosylation at position 226 of the amino acid sequence of preS/S gene.

### 3.6 Secondary structures prediction

**Figure 3:**
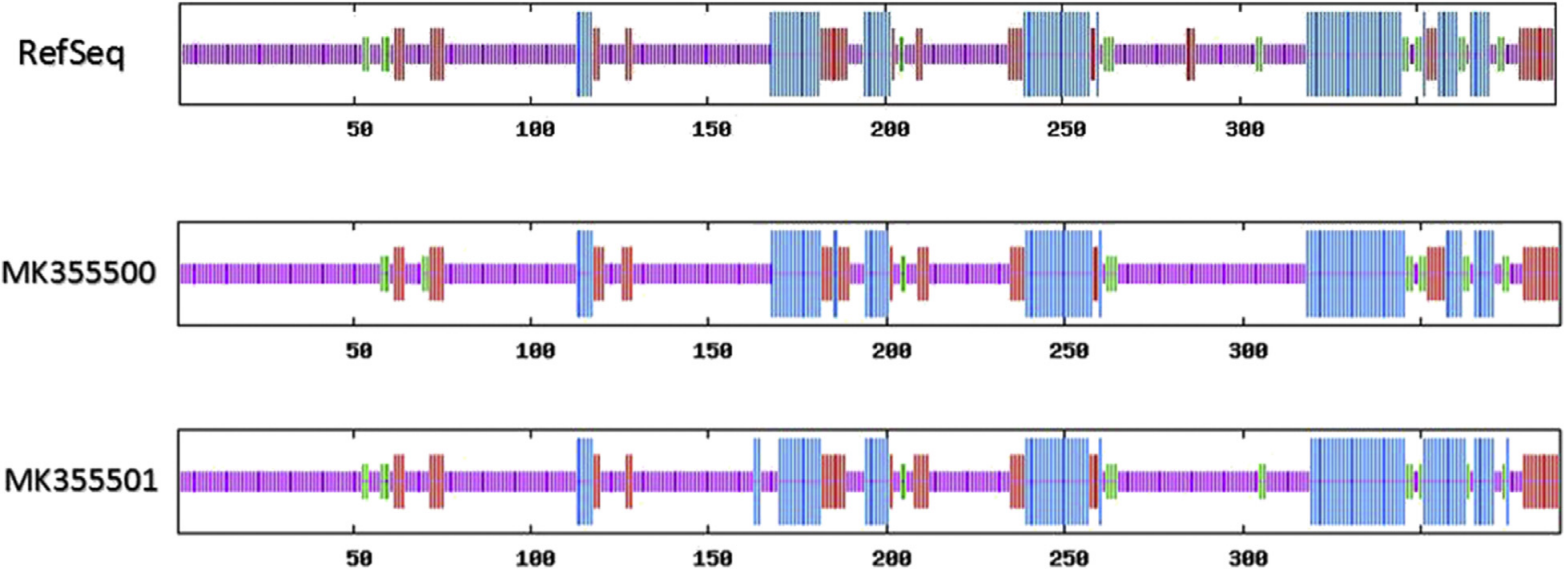
Secondary structure prediction for Ahvaz sequences and RefSeq. Blue is alpha helix; green is beta turn; purple is random coil and red region is extended strand.

The results of SOPMA and GAMMAPred methods are summarized in Table 6. The schematic exhibition of proteins secondary structure is shown in Figure 3. According to these results, the most percentage of secondary structures of S protein belonged to random coil.

**Table 6:** Secondary structure percentage using SOPMA software.

Sequences	Alpha helix %	Beta turn %	Extended strand %	Random coil %
RefSeq	22.37%	5.40%	11.31%	**60.93%**
MK355500	22.37%	5.14%	11.31%	**61.18%**
MK355501	23.39%	4.63%	10.28%	**61.70%**

The results showed that most of the structure was the random coil.

### 3.7 Tertiary structures prediction and validation

**Figure 4:**
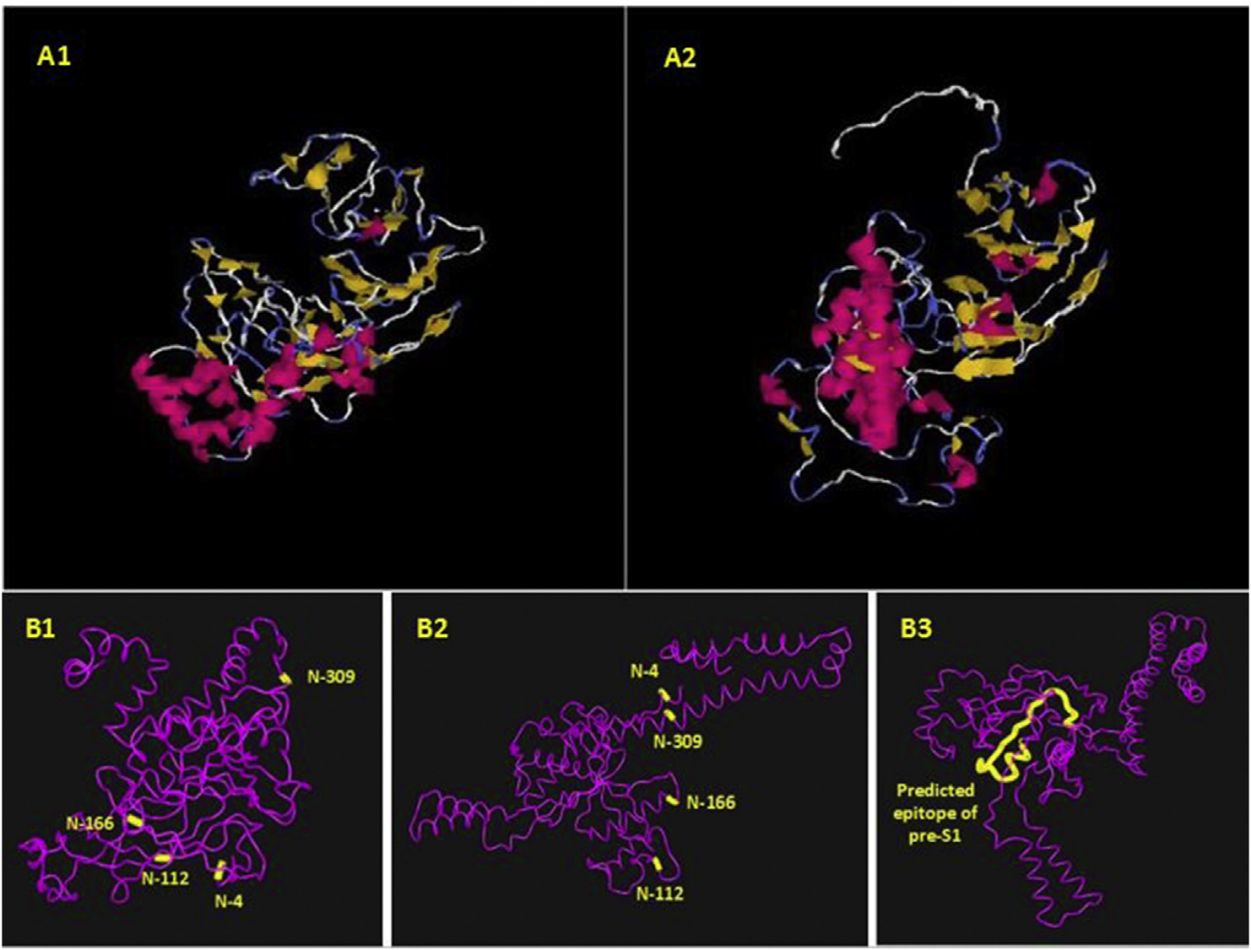
Prediction of the tertiary structure using I-TASSER online software for Ahvaz sequences. A. Results of our study revealed that I-TASSER could create more reliable 3D structures compared to other software (A1: 3D structure of MK355500, A2: 3D structure of MK355501). Pink is alpha-helix; yellow is beta-sheet; blue is coil and white region is extended strand; and B. Positions of N-glycosylation sites (B1: MK355500 isolate, B2: MK355501 isolate) and predicted B cell epitope (B3: predicted epitope of pre-S1 (MK355501 isolate)). The selected epitope starts from amino acid (21–45) which is marked in yellow.

**Figure 5:**
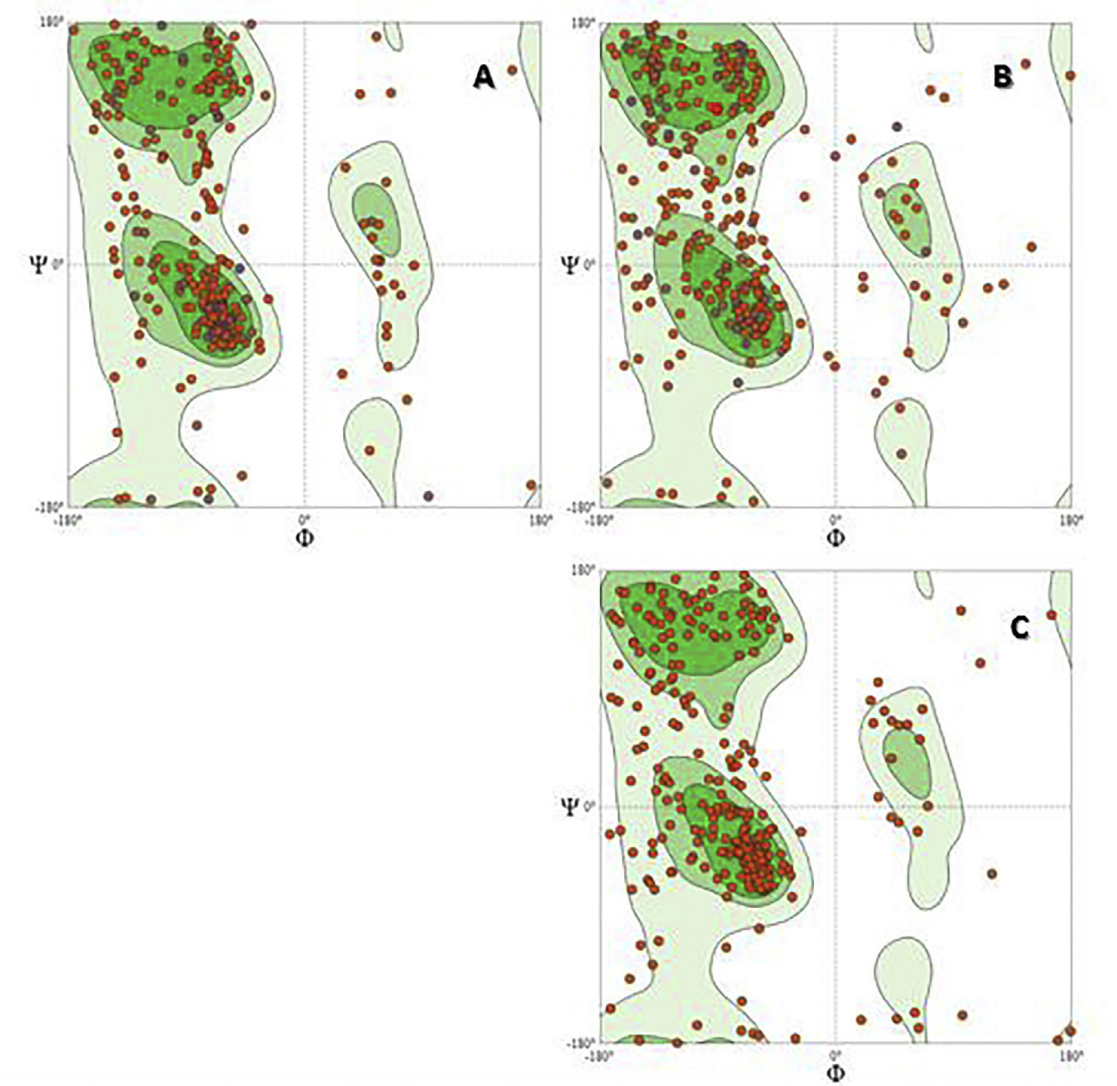
Ramachandran plot results for tertiary structure of S protein using Swiss-model online software. The results revealed that high amino acids were located at favored regions (69.2%) while low amino acids (19.6%) were distributed in the allowed region. (A: MK355500, B: MK355501, and C: RefSeq).

The 3D structures of our sequences for preS/S gene were predicted by I-TASSER, Phyre2server and (PS)_2_–V2 online software, and the results of each software were evaluated by Q-mean and Rampage software. The analysis of tertiary Structures Prediction and Validation shown by I-TASSER, found a better method for the prediction of the 3D structure of the preS/S gene (Figure 4). Q-score and Z-score for 3D structures of preS/S gene were -3.06 and 1.77, respectively. Ramachandran plots by Swiss-Model server for 3D-structure are shown in Figure 5. To confirm the predicted 3D structures by I-TASSER, Rampage software was used. The means of residues in allowed region and residues in favoured region for I-TASSER were 76 (19.6%) and 268 (69.2%), respectively.

Using iCn3D online software [51], the positions of N-glycosylation sites and predicted B cell epitope of MK355500 and MK355501 isolates are shown in Figure 4 (B1–B3).

## 4 Discussion

Bioinformatics tools are effective ways used for analysis, examination, and prediction of biological events. In recent years, the various bioinformatics tools have been developed but necessarily validation experiments need to be accomplished [39]. This research is a comparative analysis of HBV preS/S gene extracted from two chronic patients with HBV in Ahvaz and RefSeq strains of genotype D. The outcomes revealed the presence of several amino acid mutations at positions 24, 31, 41, 46, 49, 68, 76, 77, 87, 92, 109, 126, 142, 163, 169, 186 and 207 in our sequences of S gene related to alignment tree. These mutations of amino acids could be relevant to diversity in epitopes, antigenicity, secretion, and expression of the S gene [52]. The obtained data will be useful for further analyses like expression, cloning, and purification of S gene.

Previous studies realized that mutations at amino acid positions 126, 129, 130, 135, 136, 141, 144 and 145 of ‘a’- determinant resulted in non-detectable HBsAg in samples [53,54]. In this study, T126N mutation was observed in one of HBV isolates (MK355501) which may lead to the failure of HBsAg detection by ELISA tests. G145R is a well-known immune escape mutation which results in the lowest capacity of binding to all monoclonal antibodies [54]. In contrast, the G145R mutation of S gene was not observed in our sequences.

In fact, concerning our sequences, several immune epitope mutations including S45T, T113S, N131T, I194V and P142L in MK355500 and S207N in MK355501 were detected in S gene. Characterization of these sequences is required to investigate their roles in HBV infection. Lin et al. (2013) have similarly detected such mutations (S45 T/A, N131T, I194V, and S207N) in the S region [55] and recommended further research in this domain. According to the results obtained from the Geno2pheno HBV software, both sequences of Ahvaz were sensitive to anti-HBV drugs including Adefovir, Entecavir, Tenofovir, and Telbivudine. No escape mutations were found in both detected S genes. The prediction result of pI exhibited preS/S protein was an alkaline (or basic) protein. The half-life of stableness of the recombinant protein in Escherichia coli as a competent host was more than 10h. Our sequences of preS/S gene with instability index around 54–58 were identified as unstable proteins. Based on Aliphatic index, the result of preS/S gene showed the range of 78.95–82.24 and revealed that our proteins were thermally stable. In this study, all proteins were hydrophobic (with positive GRAVY value in the range 0.06–0.16). Results of the phylogenetic tree of the preS/S gene showed that Ahvaz isolates were genotype D1 and were clustered with other HBV genotype D isolates. Additionally, no recombination evidence was detected in the both HBV isolates.

The result of B cells epitope prediction by IEDB showed that the selected epitope of preS1 at position 21–45, PAFRANTANPDWDFNPNKDTWPDAN with the highest score was an important immunogenic region and could evoke a B cell response. Hu *et al.* (2005) have reported that the major immunogenic domain of preS1 was at position (21–59) [56]. In the present study, based on AlgPred software, both preS/S genes of HBV isolates were identified as non-allergic. Bepipred identified three conserved regions at positions 4–166, 197–227 and 272–297 in two Ahvaz isolates and RefSeq. The current vaccine against HBV infection was produced in various expression systems under various processing situations. In fact, the recombinant HBsAg in this vaccine is a protein with 14 cysteines (with a total of 226 amino acids); all epitopes are dependent on the intra- and intermolecular disulfide bond formation. HBsAg maturated by the formation of disulfide bonds whose complexity and heterogeneity are necessary for the formation of the neutralizing epitope like a native virus [57]. The results of DiANNA for preS/S gene exhibited 14 cysteines at positions 211, 228, 232, 239, 253, 270, 284, 287, 300, 301, 302, 310, 312 and 384 and 7 disulfide bonds. The presence of cysteines and disulfide bonds are essential for the formation of the surface antigen epitopes of HBV [58]. Both HBV isolates composed of 8 cysteines at positions 287, 300, 301, 302, and 310 and were located in the ‘a’ determinant of S gene (corresponding to sites 124, 137, 138, 139, and 147 at S protein). The results of phosphorylation sites by DISPHOS software showed that there was a large number serine residues (around 30–33) for Ahvaz sequences but none of them were considered as a phosphorylation site. The immunodominant a-determinant located at the S gene of HBV was the major target of neutralizing antibodies and the crucial determinant for infectivity. N-glycosylation site occurred at position 146 of this region and affected the function of S protein. Julithe *et al.* (2014) determined that glycosylated N146 at the S protein displays the dual function in infectivity and immune escape [59]. In this study, analysis of glycosylation by NetNGlyc software revealed that there were four conserved residues, (4, 112, 166 and 309) for N-glycosylation. Two of N-glycosylation (166 and 309) sites were detected within S gene among which only N-glycosylation site at position 309 was located in ‘a’ determinant region (corresponding to N146 at S gene). Yu *et al.* (2014) reported that the occurrence of N-glycosylation mutations in HBsAg MHR resulted in the decreased affinity to anti-HBs antibody and enhanced virion release. These results may lead to increased virus infectivity and immune escape [60]. In this study, no mutation in amino acid N146 was detected in the S protein of both HBV isolates. To precisely evaluate the analysis of disulfide bond (DiANNA and SCRATCH), phosphorylation site (DISPHOS and NetPhos), and glycosylation site (NetNGlyc and GlycoEP), positions of two isolates were compared with RefSeq strain. According to the results analysed by DiANNA and SCRATCH, the distribution of disulfide did not indicate any significant dissimilarity. Also, no significant dissimilarity between the applied methods (DISPHOS and NetPhos) and (NetNGlyc and GlycoEP) was found. Advantageously, these methods used primary sequence information and natural network to analyse the isolates from Ahvaz [61,62]. Prediction of the secondary structure of the preS/S gene revealed the highest percentages of components including the random coil, followed by an alpha helix, extended strand, and beta-turn. S region is composed of several transmembrane helices. A large number of N-glycosylation and B-cell epitopes sites were located in the random coil. The distribution of disulfide bonds were found in amino acids at 211, 239, 253, and 384 in the extended strand region and also amino acids at positions 228, 232, 270, 284, 287, 300, 301, 302, 310, and 312 in the random coil region. The results of tertiary structure prediction by I-TASSER, Phyre2 and (PS)_2_–V2 software confirmed that I-TASSER software was the most reliable tool for prediction of 3D-structure of preS/S gene. Ramachandran plot revealed that most residues were placed in the favoured regions rather than allowed regions.

## 5 Conclusion

This research attempted to investigate several new substitution sites (T126N, N131T, P142L, and S207N) identified in two HBV S proteins. It was observed that the majority of these sites were present in ‘a’ determinant region of S gene and may result in the failure of detection HBsAg in patient's samples by ELISA tests. In addition, 14 cysteines and 7 disulfide bonds were detected in both HBV isolates. Prediction of 4 N-glycosylation sites at positions 4, 112, 166, and 309 exhibited that only site 309 (corresponding to 146 at S gene) was located in ‘a’ determinant region. No escape mutation was observed in the preS/S gene of both HBV isolates. The phylogenetic tree showed that our preS/S gene of two HBV isolates were genotype D1 and were clustered with other HBV genotype D isolated from different regions of the world. Moreover, a selected epitope of preS1 gene at position 21–45, (PAFRANTANPDWDFNPNKDTWPDAN) was found as highly immunogenic B cell epitope for two isolates. Overall, the outcomes of this study are of great value for the better comprehension of the preS/S structural analysis, HBV infection and designing an effective epitope for developing vaccine.

## Declarations

### Author contribution statement

Nastaran Khodadad: Performed the experiments; Analyzed and interpreted the data; Contributed reagents, materials, analysis tools or data; Wrote the paper.

Seyed Saeed Seyedian, Afagh Moattari: Conceived and designed the experiments; Contributed reagents, materials, analysis tools or data.

Somayeh Biparva Haghighi: Contributed reagents, materials, analysis tools or data; Wrote the paper.

Roya Pirmoradi: Performed the experiments; Analyzed and interpreted the data.

Samaneh Abbasi: Contributed reagents, materials, analysis tools or data.

Manoochehr Makvandi: Conceived and designed the experiments; Analyzed and interpreted the data; Wrote the paper.

### Funding statement

This work was supported by the Infectious and Tropical Diseases Research Center, Health Research Institute of Ahvaz Jundishapur University of Medical Sciences, Ahvaz, Iran.

### Competing interest statement

The authors declare no conflict of interest.

### Additional information

No additional information is available for this paper.

## Appendix A Supplementary data

Supplementary_V3 Supplementary_V3
