# Supplementary material for: In silico functional and structural characterization of hepatitis B virus PreS/S-gene in Iranian patients infected with chronic hepatitis B virus genotype D
Source: Heliyon. 2020 Jul 15;6(7):e04332. doi: 10.1016/j.heliyon.2020.e04332 (PMC7365991; doi:10.1016/j.heliyon.2020.e04332)
Supplement: Supplementary_V3 [file mmc1.docx]

**Supplementary Material**

**Table I.** Primers used for sequencing of Ahvaz isolates

| Name of primers | Sequences 5ʹ-------> 3ʹ |
| --- | --- |
| M13-F | TGTAAAACGACGGCCAG |
| M13-R | CAGGAAACAGCTATGAC |
| SB1 | TTAGGGTTTAAATGTATACC |
| P1 | TCACCATATTCTTGGGAA |

**Table II.** List of all sequences was used in study and 2 Ahvaz sequences for analysis of bioinformatics. NC003977.1 is also used as reference sequence (RefSeq).

| Sequences marked with Accession number | | | |
| --- | --- | --- | --- |
| **MK355500** | JX661477.1 | AY741797.1 | JQ927384.2 |
| **MK355501** | EU579443.1 | AY741796.1 | HE815465.1 |
| GQ184322.1 | FJ562338.1 | AY741795.1 | AM282986.1 |
| KF584166.1 | EU579442.1 | AY741794.1 | AB583680.1 |
| HE981176.1 | HE576989.1 | AB485808.1 | KT963508.1 |
| FJ356716.1 | KC875340.1 | AP007262.1 | AB126581.1 |
| HE981181.1 | KF471640.1 | AB205010.1 | AB036920.1 |
| EU579441.1 | AY741798.1 | AB486012.1 | AB368295.1 |

**Table III.** Amino acid changes in Ahvaz isolates and RefSeq using by Geno2pheno online software.

| **Sequences** | | **Mutations** |
| --- | --- | --- |
| RefSeq | ***LHB*** | T14Q, V18T, P19S, G35R, S38T, N39A, I48N, H51T, A54D, Q57K, V60A, P65L, V74L, T86Q, V88L, S89P, T90A, I91N, I108L, D114N, S115T |
|  | ***MHB*** | A7T, A11T, K16R, A35V, P36L, N37T, I38T, H41P, I42L, S46F, A47S, T49I, V53A, T54L |
|  | ***SHB*** | S45T, P46T, I68T, T113S, T114S, K122R, T125M, P127T, N131T, F134Y, A159G, Y161F, V168A, A194V, V209L, I213L |
| MK355500  (Positive for HBsAg) | ***LHB*** | T14Q, V18T, P19S, D31G, G35K, S38T, N39A, I48N, H51T, A54D, Q57K, V60A, P65L, V74L, G76K, W77R, T86Q, V88L, S89P, I91N, I108L, D114N, S115T |
|  | ***MHB*** | A7T, A11T, K16R, A35V, N37T, I38T, A39V, S46F, A47S, T49I, V53A, T54L |
|  | ***SHB*** | S45T, P46T, I68T, L87P, L109R, T113S, T114S, K122R, N131T, F134Y, P142L, A159G, Y161F, W163R, V168A, A194V, V209L, I213L |
| MK355501  (Negative for HBsAg) | ***LHB*** | T14Q, V18T, P19S, G35R, S38T, N39A, I48N, H51T, A54E, Q57K, V60A, P65L, T68P, V74L, T86Q, V88L, S89P, I91N, I108L, D114N, S115T |
|  | ***MHB*** | A7T, A11T, K16R, A24V, A35V, N37T, I38T, A39V, H41P, A47S, V53A, T54L |
|  | ***SHB***  ***SHB*** | S45T, P46T, Q54R, I68T, I92T, T113S, T114S, K122R, T126N, N131T, F134Y,  A159G, Y161F, V168A, R169P, L186H, A194V, S207N, V209L, I213L |
